# *Post-loss speeding* or *post-win slowing*? An empirical note on the interpretation of decision-making time as a function of previous outcome

**DOI:** 10.3758/s13423-024-02460-0

**Published:** 2024-03-04

**Authors:** Benjamin James Dyson

**Affiliations:** 1 Department of Psychology, University of Alberta, Edmonton, P-217 Biological Sciences Building, Edmonton, AB T6G 2E9 Canada; 2 Toronto Metropolitan University, Toronto, ON Canada

**Keywords:** Post-loss slowing, Post-loss speeding, Post-win slowing, Post-win speeding, Post reinforcement pause

## Abstract

Differences in response time following previous losses relative to previous wins are robust observations in behavioural science, often attributed to an increased (or decreased) degree of cognitive control exerted after negative feedback, hence, *post-loss slowing (*or *post-loss speeding).* This presumes that the locus of this effect resides in the specific modulation of decision time following negative outcomes. Across two experiments, I demonstrate how the use of absolute rather than relative processing speeds, and the sensitivity of processing speeds in response to specific experimental manipulations (Experiment 1: win rate, Experiment 2: feedback), provide clarity as to the relative weighting of *post-win* and *post-loss* states in determining these behavioural effects. Both experiments show that the speeding or slowing of decision-time is largely due to the flexibility generated by *post-win* cognitive states. Given that *post-loss speeding* may actually represent *post-win slowing*, conclusions regarding the modulation of decision-making time as a function of previous outcomes need to be more carefully considered.

## Introduction

*Post-loss speeding* and *post-win slowing* are central findings in the cognitive control literature implying the acceleration or deceleration, respectively, of reaction time following the commission of an incorrect response (e.g., Pfister & Foerster, 2022). The observation of speeding or slowing is determined by both the nature of the task, and the proficiency with which the task is completed. For example, experimental psychology tasks tend to produce *post-loss slowing* whereas simulated gambling paradigms tend to produce *post-loss speeding* (Eben et al., 2022). One reason for this is the difference in proficiency exhibited in each case: experimental psychology emphasizes accuracy (and hence a high degree of success) whereas gambling environments are designed to keep player success low.

Dyson et al. (2018) showed that both *post-loss speeding* and *post-loss slowing* could be observed within the same paradigm, where the direction of the effect was determined by the degree of wins and losses exhibited. Specifically, playing Rock, Paper, Scissors against a computerized opponent who could not be exploited (i.e., win rate was low), *post-loss speeding* was expressed. However, when participants were able to exploit their computerized opponent (i.e., win rate was high), *post-loss slowing* was expressed (Dyson et al., 2018). Both observations are consistent with an orienting account by Notebaert et al. (2009, p. 275) whereby “infrequent events orient attention away from the task.” Given the perceptual nature of the Notebaert et al. (2009) paradigm, responses resulting in the presentation of incorrect feedback were classed as *errors* (rather than losses) but the same conclusion holds. If correct responses are frequent and incorrect responses are infrequent, then *post-error slowing* will result due to the rarity of incorrect responding. In contrast, *post-error speeding* will result if the incorrect responses exceed the number of correct responses (see also Notebaert et al., 2009, Experiment 1). For the remainder of the paper, I use the term *post-loss* to incorporate findings from both *post-error* and *post-loss* literatures.

Despite the resolution of *post-loss* speeding and *post-loss* slowing as a function of outcome frequency, what remains curious, however, is the implicit insistence that the locus of this effect is the degree of cognitive control exerted post-*loss*. For example, Dutilh et al. (2012), p. 1) discuss the resultant slowing in terms of “increased response caution” following negative feedback, whereby greater control (or perceived control; Eben et al., 2022; Eben et al., 2023) over responding increases the likelihood of correct responding on the following trial (see also Botvinick et al., 2001). This characterization of *post-loss speeding* or *post-loss slowing* implies that decision-making is under volitional control following negative outcomes. In other words, individuals are better able to modulate the subsequent speeding or slowing of their behavior after receiving negative feedback. However, this appears to be in contrast with several observations in the literature.

First, the default observation of *post-loss speeding* in contexts where individuals experience an equivalent degree of wins and losses (e.g., Dyson et al., 2018) is consistent with the initiation of more intuitive and inflexible action following negative outcomes, relative to more rational and flexible decisions following positive outcomes (i.e., System 1 rather than System 2; Kahneman, 2011). Second, other studies have shown that neural signals generated by negative feedback (e.g., feedback-related negativity; FRN) modulate less than the FRN generated by positive feedback (Cohen et al., 2007; Forder & Dyson, 2016), revealing a possible neurological origin of the inflexible behaviour observed down-stream after negative feedback. Therefore, the first step in adjudicating whether losses produce flexible cognitive control allowing for variable speeding and slowing is to examine the degree to which *post-loss* behaviours are sensitive to experimental manipulation. My logic is that if *post-loss* behaviour is less sensitive to experimental manipulation relative to *post-win* behaviour, then this is a further indication the former in less under cognitive control and, thus, less likely to be responsible for the origin of speeding and slowing.

Problems in disambiguating the origin of decision-making speeding or slowing are also compounded by the traditional ways in which *post-loss speeding* or *post-loss slowing* measures are calculated. For example, Dutilh et al., ( 2012, Table 1) summarize the standard way to assess speeding or slowing as calculating the difference between average reaction time *post-error* minus average reaction time *post-correct* for each participant. Therefore, positive values index *post-loss slowing* (such as the +25ms observed in the 75% accuracy condition of Notebaert et al., 2009, Experiment 1) whereas negative values index *post-loss speeding* (such as the -59 ms observed in the 35% accuracy condition of Notebaert et al., 2009, Experiment 1). Unfortunately, the use of a difference score obfuscates exactly what is speeding or slowing. Additional approaches to measurement such as estimating the variance in reaction time following loss, or, calculating the difference between *post-loss* and *pre-loss* trials (Dutilh et al., 2012) are similarly uninstructive in testing a central assumption in the so-called *post-loss* literature: that the speeding and / or slowing is the result of variable cognitive control following losses (although see Rabbitt, 1966, for a discussion of other factors). Therefore, the second step in adjudicating whether losing leads to flexible cognitive control allowing for variable speeding and slowing is to examine absolute *post-loss* and *post-win* response times (RTs) rather than the relative difference between RTs.

**Table 1:** Descriptive and main inferential statistics for Experiments 1 and 2

Descriptive statistics					
Experiment 1	25% Win		50% Win	75% Win	
* Post-Win*	667 (64)		597 (54)	453 (32)	
* Post-Loss*	500 (43)		475 (44)	422 (25)	
Experiment 2	Feedback		No Feedback		
* Post-Win*	671 (58)		515 (32)		
* Post-Loss*	525 (33)		530 (35)		
Inferential statistics					
Experiment 1	df	F	MSE	p	ƞ_p_^2^
*** Win Rate (W)***	**2,190**	**7.09**	**149093**	**.001**	**.069**
*** Outcome (O)***	**1,95**	**38.22**	**42005**	**<.001**	**.287**
*** W x O***	**2,190**	**12.25**	**41129**	**<.001**	**.114**
Experiment 2	df	F	MSE	p	ƞ_p_^2^
* Feedback (F)*	1,71	3.06	132367	.085	.040
*** Outcome (O)***	**1,71**	**10.34**	**29644**	**.002**	**.127**
*** F x O***	**1,71**	**9.08**	**51191**	**.004**	**.113**

Standard error in parenthesis. Bold terms indicate statistical significance

To examine the contribution(s) of putatively negative (*post-loss)* and positive (*post-win)* feedback in producing effects traditionally attributed to *post-loss* states, I examined the sensitivity of absolute *post-loss* and *post-win* decision time in response to two manipulations: win rate (Experiment 1), and, the presence or absence of feedback (Experiment 2). The justification for the win rate manipulation in Experiment 1 was based on previous work whereby decision-making speeding and slowing was contingent on outcome frequency (e.g., Dyson et al., 2018; Notebaert et al., 2009). The justification for the feedback manipulation in Experiment 2 was also based on previous work where weakening the strength of association between trials resulted in the modulation of *post-win* trials but not *post-loss* trials (e.g., Srihaput et al., 2020). In both cases, a schematic will help to disambiguate the relative contributions of both *post-loss* and *post-win* in observing these effects (see also Kerzel et al., 2022, for a similar logical argument for statistical learning effects in visual search).

## Experiment 1

**Fig. 1:**
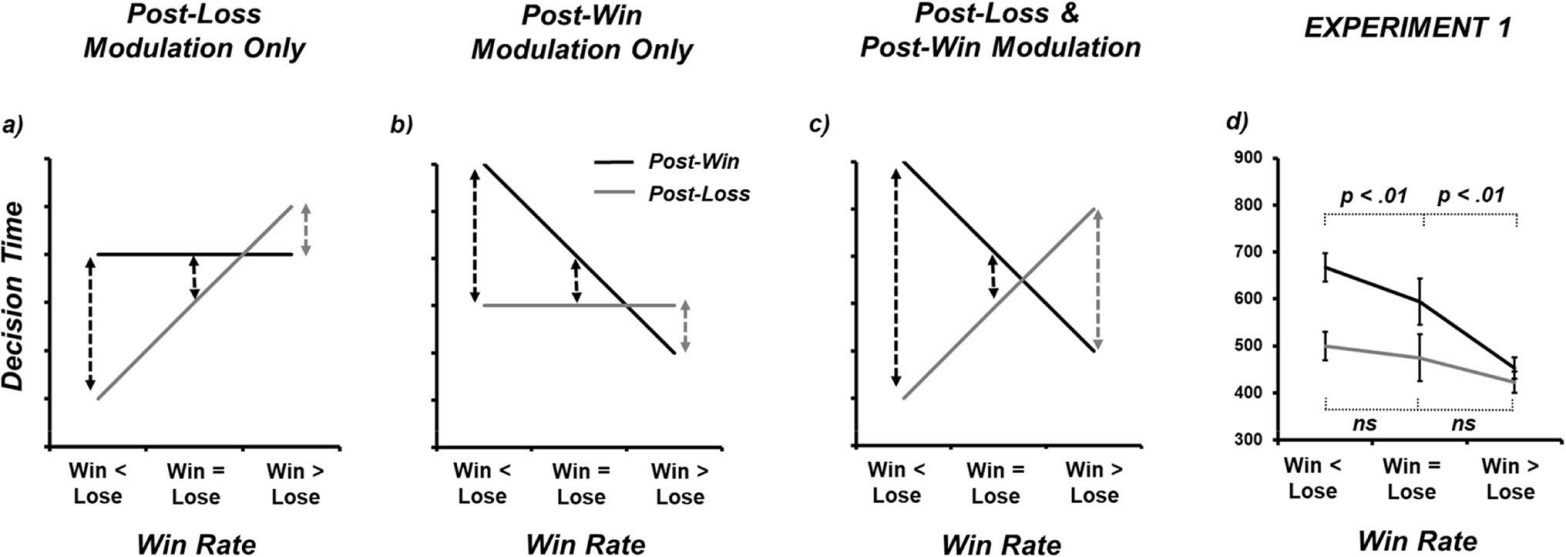
Schematic data in support of three different expression of traditional ‘*post-error speeding*’ effects when wins are equal to (or exceed) losses (represented by black dotted arrows), and ‘*post-error slowing*’ effects when losses exceed wins (represented by grey dotted arrows). (**a**) Effects due to the modulation of *post-loss* speed only. (**b**) Effects due to the modulation of *post-win* speed only. (**c**) Effects due to the modulation of both *post-loss* and *post-win* speed. (**d**) Empirical data showing trial *n+1* decision time (ms) as a function of win rate condition (25% [*win < lose*], 50% [*win = lose*], 75% [*win > lose*]) and trial outcome at trial *n* (*win, loss*). At the request of a reviewer, within-participant standard error of paired differences for each pairwise comparison of *post-win* and *post-loss* trials are plotted (Pfister & Janczky, 2013). Decision-time data produced a pattern similar to Fig. 1b, confirming that the volitional control of decision-making time occurs following positive rather than negative outcomes

I began my interpretative logic by considering a hypothetical case representing the long-run equivalence of positive and negative outcomes (*win* = *lose*). On the basis of previous work where individuals experience an equivalent degree of wins and losses (e.g., Dyson et al., 2018), *post-loss* decisions (in grey) should take less time than *post-win* decisions (in black). This difference between *post-win* and *post-loss* decision time is further represented by a black dotted line, indexing that decision times following wins are longer than decision times following losses. Collectively, these data are traditionally interpreted as ‘*post-loss speeding*’ and are represented in the middle column of Fig. 1a. The other two columns of Fig. 1a simulate the generation of additional empirical effects by *only* modulating *post-loss* decision times. Specifically, an exacerbation of *post-loss speeding* when loss rates exceed win rates (*win < lose;* as per Notebaert et al., 2009), and, a switch from *post-loss speeding* to *post-loss slowing* when win rates exceed loss rates (*win > lose*; as per Dyson et al., 2018). When decision times following losses are longer than wins, this is represented by a grey dotted line. Thus, Fig. 1a provides a hypothetical account of *post-win* and *post-loss* decision time effects, whereby the flexible cognitive control that allows for variable speeding and slowing is only present in *post-loss* responding.

Moving to Fig. 1b, exactly the same differences between *post-win* and *post-loss* decision times are represented: the standard case of decision times following wins being longer than decision times following losses when *win = loss* (middle black dotted line), an exacerbation of this effect when *win < loss* (left-most black dotted line), and, a reversal of this effect when *win > loss* (right-most grey dotted line). However, the locus of these effects in Fig. 1b cannot be attributed to the modulation of decision times *post-loss* as these are now stable across all three conditions. Rather, these effects are now being generated *only* by the modulation of *post-win* decision times. Under this hypothetical account, data are more accurately interpreted as *post-win slowing* for *win < lose* and *win = lose* conditions (rather than the inaccurate label of *post-loss speeding*), and, *post-win speeding* for *win > lose* conditions *(*rather than the inaccurate label of *post-loss slowing)*. This is because it is the *post-win* state that generates the changes in decision time, not the *post-loss* state.

A final Fig. 1c represents a third hypothetical possibility where both positive and negative outcomes contribute to the modulation of decision time on the next trial. To foreshadow my results, RT data produced a pattern similar to Fig. 1b, identifying that the volitional control of decision-making time occurred following positive (*post-win*) rather than negative (*post-loss*) outcomes.

### Method

Ninety-six individuals were analyzed from the undergraduate community at the University of Alberta as part of the Psychology Research Participation scheme, and received performance-independent course credit (Research Ethics Board 2; Pro00116715). In both Experiments 1 and 2, the rationale for the final sample size was determined by counterbalancing requirements and the time constraints associated with in-person undergraduate data collection. An initial set of 14 participants who ran in the ‘odd sum’ version of Experiment 1 were replaced, due to the presence of an additional blank screen that was absent in the ‘even sum’ version (see below). There were no exclusions on the basis of data quality. Of the participants who provided optional demographic information (81), 55 identified as female and 26 identified as male, and the mean age was 18.85 years (SD = 1.82). Images of the individual sides of a six-sided dice were used: white spots on a black background representing participant choices, and, black spots on a white background representing opponent choices. Stimulus presentation and response recording were coordinated through Presentation 23.0 (build 10.27.21; Neurobehavioral Systems Inc., Albany, CA, USA). At the end of the study, participants also completed the BIS-BAS questionnaire (Carver & White, 1994), the data for which will not be discussed here.

Participants completed 480 trials of a novel game *Dice Dual* (based on Hayes, 1975), divided into three conditions defined by fixed win rate (25% [*win < lose*], 50% [*win = lose*], 75% [*win > lose*]; 160 trials each). The order of the 25% and 75% conditions were counterbalanced around the 50% condition (25–50–75%, or, 75–50–25%). Prior to each condition, participants were reminded of the rules of the game. At each trial, participants selected one of the 6 die sides and their computerized opponent did the same. For half of the participants, the rule was that if the sum of both dice was even they would win and if the sum of both dice was odd they would lose. This rule was reversed for the other half of the participants. In all cases, winning resulted in the sum of both dice being added to the participant’s total, whereas losing resulted in the sum of both dice being subtracted from the participant’s total.

Win rates were fixed within each condition in that the outcome of each trial was predetermined and the computer randomly selected a response from the category that would guarantee the required outcome. To provide one specific example, the winning rule is to have an even sum and the trial is predetermined as a *participant win* trial. If the participant selected 3, the computer would randomly select another odd number (1, 3, 5) yielding the sum of 4, 6 or 8 (all even), with this sum subsequently added from the participant’s score and subtracted from the computer’s score. Following participant response selection, both dice were presented for 1,000 ms (opponent on left, participant on right) with the sum of the dice centre screen. This was replaced by the outcome ‘WIN (+x)’ in green or ‘LOSE (-x)’ in red, with x representing the sum of both dice. Opponent and participant scores (bottom left and bottom right, respectively) would be updated for 1,000 ms. The next trial would begin with the presentation of the participant’s six die sides again.

### Results

To pre-empt the exploratory analyses of Experiment 1 (see Fig. 1d), decision times were decreased following losses relative to wins, and, decreased as the frequency of win rate increased. An interaction between win rate and outcome showed that the modulation of decision-time was largely due to *post-win* responses, most consistent with the schematic depicted in Fig. 1b.

To check the effectiveness of my win rate manipulation in Experiment 1, average dice value at trial *n+1* was assessed as a function of win rate condition (25%, 50%, 75%) and trial outcome at trial *n* (*win, loss*) via a two-way repeated-measures ANOVA. The main effect of win rate (F[2,190] = 7.04, MSE = 0.703, *p* = .001, ƞ_p_^2^ = .069) showed that participants selected higher values as win rate increased (25% = 3.92; 50% = 4.04; 75% = 4.24), although only the 75% win rate condition was significant from all other conditions (Tukey’s HSD, *p* < .05). The main effect of outcome (F[1,95] = 13.41, MSE = 0.077, *p* < .001, ƞ_p_^2^ = .123) showed that participants selected higher values *post-win* relative to *post-loss* (4.11 vs. 4.03). There was no significant interaction between win rate × outcome (F[2,190] = 0.71, MSE = 0.055, *p* = .493, ƞ_p_^2^ = .007). Therefore, participants were more risky following wins relative to losses, and, in the *win > lose* condition.

Median RTs at trial *n+1* as a function of win rate condition (25%, 50%, 75%) and trial outcome at trial *n* (*win, loss*) for each participant were run through a two-way repeated-measures ANOVA (see Table 1 and Fig. 1d). Main effects of win rate (F[2,190] = 7.09, MSE = 149093, *p* = .001, ƞ_p_^2^ = .069) and outcome (F[1,95] = 38.22, MSE = 42005, *p* < .001, ƞ_p_^2^ = .287) were subsumed in a two-way interaction between win rate × outcome (F[2,190] = 12.25, MSE = 18929, *p* < .001, ƞ_p_^2^ = .114; see Fig. 1d). The main effect of win rate showed that RT decreased as the frequency of wins increased (25% wins = 583 ms, 50% wins = 534 ms, 75% wins = 438 ms), with the 75% win rate condition significantly different from both 25% and 50% win rate conditions (Tukey’s HSD, *p* < .05). The main effect of outcome showed that RT were overall decreased for losses (466 ms) relative to wins (571 ms).

The critical interaction between win rate × outcome was due to the significant slowing of RT as a function of win-rate RT reduction *post-win* (25% = 687 ms; 50% = 604 ms; 75% = 506 ms; all Tukey’s HSD p < .05) in contrast to the weaker win-rate RT modulation *post-loss* (25% = 503 ms; 50% = 482 ms; 75% = 466 ms; all Tukey’s HSD *p* < .05, apart from 25% vs. 75%). These data clearly favour the interpretation of post-win slowing (as opposed to post-loss speeding) given the flexibility of RTs following wins relative to the inflexibility of RTs following losses (see schematic Fig. 1b). For Experiment 1, the post-win RT difference across the three levels of fixed win rate effect size was estimated as 1.576 ([CI -/+ 95%: 1.325 – 1.824]; *n* = 96; Cohen’s *f*).

## Experiment 2

**Fig. 2:**
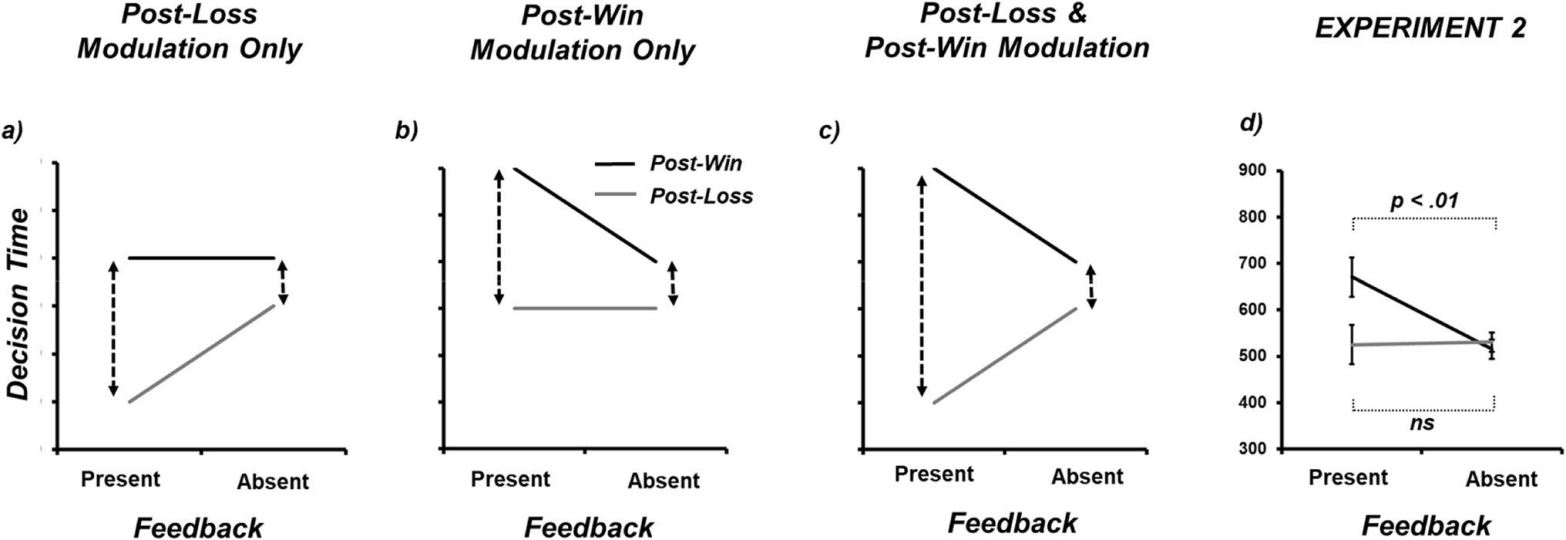
Schematic data in support of three different expression of traditional ‘*post-error speeding*’ effects (represented by black dotted arrows) and ‘*post-error slowing*’ (represented by grey dotted arrows) in response to the presence or absence of direct feedback. (**a**) Effects due to the modulation of *post-loss* speed only. (**b**) Effects due to the modulation of *post-win* speed only. (**c**) Effects due to the modulation of both *post-loss* and *post-win* speed. (**d**) Empirical data showing trial *n+1* decision time (ms) as a function of the presence or absence of direct feedback and trial outcome at trial *n* (*win, loss*). At the request of a reviewer, within-participant standard error of paired differences for each pairwise comparison of post-win and post-loss trials are plotted (Pfister & Janczky, 2013). Decision-time data produced a pattern similar to Fig. 1b, confirming that the volitional control of decision-making time occurs following positive rather than negative outcomes. At the request of a reviewer, within-participant standard error of paired differences of *post-win* and *post-loss* trials are plotted (as per Pfister & Janczky, 2013). Raw standard errors are provided in Table 1

To test the generalizability of the results from Experiment 1, I moved from a six-response, two-outcome game (*Dice Dual*) to a three-response, three-outcome game (*Rock, Paper, Scissors; RPS*). In Experiment 2, I examined the sensitivity of *post-loss* and *post-win* RT in response to a different experimental manipulation: the presence or absence of direct feedback (‘win’, ‘loss’ or ‘draw’). In the case of RPS, presenting both player and opponent responses prior to the delivery of ‘win’, ‘loss’ or ‘draw’ may allow individuals to resolve the outcome of the trial based on *indirect feedback* (e.g., playing Paper against Scissors will result in a loss; see Forder & Dyson, 2016, for a discussion). This is in contrast to a more potent delivery of feedback combining both *indirect* followed by *direct feedback* (‘win’, ‘loss’ or ‘draw’) regarding the outcome of the trial. Previous research has shown that reducing the potency of outcome-action associations across trials (e.g., changing rather than maintaining opponents across consecutive trials; Srihaput et al., 2020) leads to modulation in *post-win* trials but not *post-loss* trials. This lack of flexibility suggests that *post-loss* behaviour is less under cognitive control and, thus, less likely to be responsible for the causing speeding and/or slowing. Therefore, in weakening the action-outcome association via indirect feedback only, this might enable a higher degree of control *post-loss* such that we see an alternative pattern of data regarding the cause of speeding and slowing. As per Experiment 1, similar predictions can be made in terms of the difference between decision times following negative and positive feedback being caused by the modulation of (a) *post-loss* only, (b) *post-win* only, or (c) both *post-loss* and *post-win* (see Figs. 2a, b, c).

### Method

Seventy-two individuals were analysed for Experiment 2 as part of the Psychology Research Participation scheme at the University of Alberta, and received performance-independent course credit (Research Ethics Board 2; Pro00120835). Seven additional participants were not included: two participants were replaced due to technical difficulties, and five extra participants represented surplus scheduling (counterbalancing required that the final sample size was a multiple of 24). There were no exclusions on the basis of data quality. Of the participants who provided optional demographic information (59), 35 identified as female and 24 identified as male, and the mean age was 19.53 years (SD = 2.31). Hardware was equivalent to Experiment 1. At the end of the study, in addition to the BIS-BAS questionnaire (Carver & White, 1994), participants also completed the BFI-11 (Rammstedt & John, 2007). These data will not be discussed.

Participants completed two counterbalanced blocks of RPS each containing 66 trials, as part of a larger study examining the relationship between decision-making heuristics during RPS and multiple-choice question completion (to be reported elsewhere). In both conditions, the computer opponent used a variant of mixed-strategy (Bi & Zhou, 2014) and played random 22 Rock, 22 Paper, and 22 Scisssors responses in random order. At each trial, participants were given the choice of Rock, Paper or Scissors, with both participant and opponent score, and the trial number presented on the bottom of the screen. After participants made their selection, a corresponding static image showed both their decision (white glove on right) and the decision of their computer opponent (blue glove on left) for 1,000 ms and then removed during a 500-ms blank screen. For the RPS block containing direct feedback, the words ‘WIN [+1]’ in green, ‘LOSE [-1]’ in red or ‘DRAW [0]’ in grey were presented for 1,000 ms. For the RPS blocks without direct feedback, the blank screen was elongated for a further 1,000 ms.

### Results

To pre-empt the exploratory analyses of Experiment 2 (see Fig. 2d), decision times were decreased following losses relative to wins. An interaction between win rate and outcome showed that the modulation of decision-time was due to *post-win* rather than *post-loss* responses, most consistent with the schematic depicted in Fig. 2b.

As an initial manipulation check for performance in Experiment 2, neither win rate during the presence of direct feedback (32.09% [SD = 5.58]; t[71] = -1.88, *p* = .064) nor during the absence of direct feedback (34.07% [SD = 5.17]; t[71] = 1.21, *p* = .229) were significantly different from the expected win rate value of 33.33%. The comparison between feedback presence and feedback absence however was significantly different as indicated by a two-sampled, repeated-measures t-test: *t*[71] = -2.15, *p* = .035.

Median RTs at trial *n+1* as a function of direct feedback (*present, absent*) and trial outcome at trial *n* (*win, loss*) for each participant were run through a two-way repeated-measure ANOVA (see Table 1 and Fig. 2d). The main effect of feedback was not significant (F[1,71] = 3.06, MSE = 132367, *p* = .085, ƞ_p_^2^ = .041). The significant main effect of outcome (F[1,71] = 10.34, MSE = 29644, *p* = .002, ƞ_p_^2^ = .127) showed that, similar to Experiment 1, RTs were overall decreased for losses (528 ms) relative to wins (593 ms). Furthermore, there was a two-way interaction between win rate × outcome (F[1,71] = 9.08, MSE = 51191, *p* = .004, ƞ_p_^2^ = .113). As in Experiment 1, the interaction was due to the absence of RT modulation *post-loss* (Tukey’s HSD *p* > .05) in contrast to the significant slowing of *post-win* RT due to the presence of direct feedback (Tukey’s HSD p < .05). Moreover, post-win RT following the presence of feedback was also significantly slower than both post-loss RTs. The data from Experiment 2 again favour the interpretation of *post-win* slowing (as opposed to *post-loss* speeding) given the flexibility of RTs following wins relative to the inflexibility of RTs following losses (see schematic Fig. 2b). For Experiment 2, the post-win RT difference between feedback present and feedback absent effect size was estimated as 0.378 ([CI -/+ 95%: 0.082 – 0.692]; *n* = 72; repeated-measures Hedges’s *g*).

## General discussion

Differences in response time following previous losses (*post-loss*) relative to previous wins (*post-win*) are robust observations in behavioural science, often attributed to an increased (or decreased) degree of cognitive control exerted after negative feedback, hence, *post-loss slowing (*or *post-loss speeding).* This presumes that the locus of this effect resides in the specific modulation of decision time following negative outcomes. To test this idea, it is important to examine (a) absolute rather than relative decision-making times following wins and losses, and (b) the degree to which *post-win* and *post-loss* time are modulated by experimental factors such as win rate (Experiment 1) or direct feedback (Experiment 2). Based on these data, I arrive at the alternative conclusion that the speeding or slowing of decision-time can be due to the flexibility generated by *post-win* cognitive states. That is, rather than describe an RT difference as *post-loss* speeding, one can describe the same effect as *post-win* slowing.

While it is important to stress that the behavioural differences generated by negative and positive feedback can originate exclusively from *post-loss* states (e.g., Eben et al., 2020; Isheqlou et al., 2022, Table 2), the question remains: what is happening *post-win* in the current data to enable this flexible responding? With respect to *post-win slowing* at least, there are connections to be made with operant conditioning and gambling literatures that reliably describe similar differences as post-reinforcement pauses (Ferster & Skinner, 1957). Here, decision time is deliberately slower to allow the player to revel in rewarding signals of success, and the positive affect this produces (see Dixon et al., 2018; Laskowksi et al., 2019). From an emotional perspective, decision-making may simply be improved by a positive affective state (for reviews, see Lerner et al., 2015; Pham, 2007). From a cognitive perspective, extending time for future decisions should also increase the likelihood of successful performance (Dyson, 2021). However, an exclusive cognitive account does not account for the maintenance of success when decisions are allocated less rather than more time (i.e., *post-win speeding*), suggesting that emotional state may be the keystone factor (Eben et al., 2020).

Despite the prevalence of two-response, two-outcome paradigms within the literature, it is important to remember that not all decision-making is binary. Although it is critical to compare unambiguously positive with unambiguously negative outcomes, the degree to which the underlying architecture of decision-making derived from this simple *win* vs. *loss* binary distinction is applicable to non-binary decision-making spaces is an open question. An obvious consideration in this regard is ambiguous outcomes such as *draw* trials like those generated in Experiment 2. The presence or absence of feedback failed to significantly modulate median RT for *post-draw* trials (537 ms vs. 505 ms, respectively; *t*[71] = 0.825, *p* = .412), thereby aligning with the data following negative (*loss*) rather than positive (*win*) outcomes. A more detailed exploration of *post-draw* behaviour in comparison to *post-win* and *post-loss* however shows a complex profile, containing some effects usually attributed to explicitly positive outcomes and some effects usually attributed to explicitly negative outcomes (Dahal et al., 2022). Furthermore, a *draw* could be considered a ‘near-win’ or a ‘near-miss’ depending on the state of the organism (West & Lebiere, 2001), emphasizing the need to consider subjective aspects of decision-making in future research.

While the current approach may help to improve the conclusions drawn from the examination of speeding and slowing effects, there are caveats and constraints. In the Introduction, I noted the empirical equivalence between the post-*error* orienting account of Notebaert et al. (2009), and, the post-*loss* slowing account of Dyson et al. (2018): if responses associated with negative feedback are infrequent, then slowing will result due to the rarity of incorrect responding. However, there may be important differences between ‘loss’ versus an ‘error’ in terms of neural signature (e.g., Gehring et al., 1993), action cancellation (e.g., Foerster et al., 2022) and attribution (e.g., Duval & Silvia, 2002). Thus, a critically important feature of design protocol is the degree to which participants have *control* over outcomes. In terms of an objective measure of control such as win rate, Dyson et al. (2018) showed that performance against an unexploitable opponent (i.e., low win rate) generated a ‘post-loss speeding’ effect whereas successful performance against an exploitable opponent (i.e., high win rate) generated a ‘post-loss slowing’ effect.

However, control is not exclusively an objective feature of an experimental paradigm. In particular, the subjective sense of control may be equally important in determining behaviour (Eben et al., 2022a, 2022b). In Eben et al. (2023), Experiment 3), participants performed variations of a color discrimination task. Two of the variations were identical in terms of sensory properties, but were differentially described to participants as ‘hard but doable’ versus ‘impossible’. Under these conditions, speeding was observed following failure in the ‘impossible’ case but slowing was observed following failure in the ‘hard but doable’ case. These data clearly show that a subjective sense of control is more likely to lead to post-loss slowing whereas the absence of a subjective sense of control is more likely to lead to post-loss speeding. Thus, it would seem essential that both objective and subjective control are considered in future work, since objective and subjective control do not always align. In the context of the current data, there are open questions regarding whether the subjective sense of control expressed via the genuine learning of an exploitative strategy (Dyson et al., 2018) is the same as the subjective sense of control generated by a high *fixed* win rate (here, Experiment 1). In conclusion, given that *post-loss speeding* may actually represent *post-win slowing*, we can begin to disambiguate conclusions regarding the modulation of decision-making time as a function of previous outcomes by considering absolute rather than relative reaction times, and, sensitivity to experimental manipulation.
